# Poly[[(μ_3_-5-amino­isophthalato-κ^3^ *O* ^1^:*O* ^3^:*N*)(1*H*-imidazole-κ*N* ^3^)zinc] 0.25-hydrate]

**DOI:** 10.1107/S1600536811050045

**Published:** 2011-11-25

**Authors:** Hai-Wei Kuai, Xiao-Chun Cheng

**Affiliations:** a Faculty of Life Science and Chemical Engineering, Huaiyin Institute of Technology, Huaian 223003, People’s Republic of China

## Abstract

In the title coordination polymer, {[Zn(C_8_H_5_NO_4_)(C_3_H_4_N_2_)]·0.25H_2_O}_*n*_, the Zn^2+^ cation has an N_2_O_2_ donor set involving two carboxyl­ate O atoms from two 5-amino­isophthalate anions, one N atom from a 5-amino­isophthalate anion, and one imidazole N atom displaying a slightly distorted tetra­hedral geometry with two additional O-atom neighbours, with Zn-to-ligand distances of 2.711 (2) and 2.717 (2) Å, respectively. Each 5-amino­isophthalate anion acts as a μ_3_-bridge linking symmetry-related Zn^II^ ions into a layered polymeric structure parallel to (100). The asymmetric unit also comprises a disordered crystal water molecule located on an inversion centre with 0.25 occupancy. In the crystal, N—H⋯O hydrogen bonds form a three-dimensional network.

## Related literature

For related structures, see: Zhang *et al.* (2007 ▶).
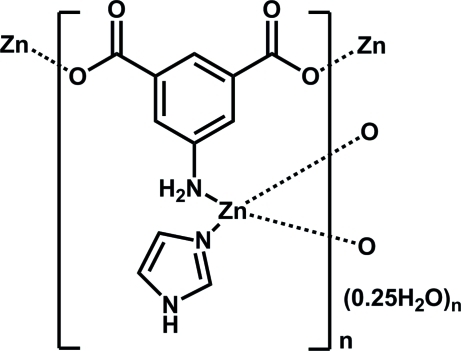


## Experimental

### 

#### Crystal data

[Zn(C_8_H_5_NO_4_)(C_3_H_4_N_2_)]·0.25H_2_O
*M* *_r_* = 317.09
Monoclinic, 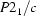

*a* = 9.6239 (11) Å
*b* = 10.1916 (11) Å
*c* = 12.1927 (13) Å
β = 95.146 (2)°
*V* = 1191.1 (2) Å^3^
*Z* = 4
Mo *K*α radiation
μ = 2.08 mm^−1^
*T* = 293 K
0.20 × 0.20 × 0.18 mm

#### Data collection

Bruker SMART APEXII CCD diffractometer
Absorption correction: multi-scan (*SADABS*; Sheldrick, 1996 ▶) *T*_min_ = 0.681, *T*_max_ = 0.706
6327 measured reflections
2340 independent reflections
1542 reflections with *I* > 2σ(*I*)
*R*_int_ = 0.038

#### Refinement

*R*[*F* ^2^ > 2σ(*F* ^2^)] = 0.048
*wR*(*F* ^2^) = 0.098
*S* = 1.09
2340 reflections
178 parameters
H-atom parameters constrained
Δρ_max_ = 0.56 e Å^−3^
Δρ_min_ = −0.57 e Å^−3^

### 

Data collection: *APEX2* (Bruker, 2008 ▶); cell refinement: *SAINT* (Bruker, 2008 ▶); data reduction: *SAINT*; program(s) used to solve structure: *SHELXS97* (Sheldrick, 2008 ▶); program(s) used to refine structure: *SHELXL97* (Sheldrick, 2008 ▶); molecular graphics: *DIAMOND* (Brandenburg, 2000 ▶); software used to prepare material for publication: *SHELXTL*.

## Supplementary Material

Crystal structure: contains datablock(s) I, global. DOI: 10.1107/S1600536811050045/kp2352sup1.cif

Structure factors: contains datablock(s) I. DOI: 10.1107/S1600536811050045/kp2352Isup2.hkl

Supplementary material file. DOI: 10.1107/S1600536811050045/kp2352Isup3.cdx

Additional supplementary materials:  crystallographic information; 3D view; checkCIF report

## Figures and Tables

**Table 1:** Selected bond lengths (Å)

N2—Zn1	1.983 (3)
O2—Zn1	1.989 (2)
Zn1—O4^i^	1.998 (3)
Zn1—N1^ii^	2.082 (3)

Symmetry codes: (i) 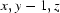; (ii) 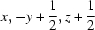.

**Table 2:** Hydrogen-bond geometry (Å, °)

*D*—H⋯*A*	*D*—H	H⋯*A*	*D*⋯*A*	*D*—H⋯*A*
N1—H1*B*⋯O4^iii^	0.91	2.21	3.018 (4)	147
N1—H1*A*⋯O2^iv^	0.88	2.17	2.943 (4)	146
N3—H3⋯O3^v^	0.95	1.88	2.822 (4)	174

Symmetry codes: (iii) 
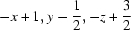
; (iv) 
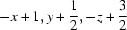
; (v) 
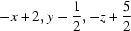
.

**Fig. 1.**
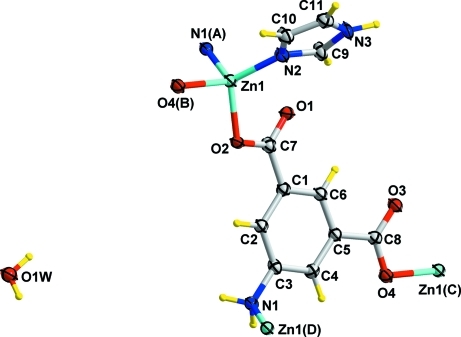
The coordination environment of zinc ion in the title complex with the ellipsoids drawn at the 30% probability level. Symmetry code used: (A) x, 1/2 - y, 1/2 + z; (B) x, -1 + y, z; (C) x, 1 + y, z; (D) x, 1/2 - y, -1/2 + z.

**Fig. 2.**
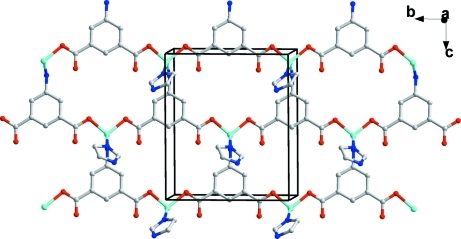
The layer built from infinite connection of zinc ions and 5-aminoisophthalate anions.

## supplementary crystallographic information

### Comment

5-Aminoisophthalic acid is often used as organic ligand to synthesise complexes with variable coordination modes. Herein, we report the crystal structure of title coordination polymer. The asymmetric unit consists of one zinc ion, one 5-aminoisophthalate anion, one imidazole and partly occupied crystal water. Each Zn ion has a N_2_O_2_ donor set and is coordinated by two carboxylate O atoms from two 5-aminoisophthalate anions, one N atom from the amino group of 5-aminoisophthalate anion, and one N atom from an imidazole, displaying a slightly distorted tetrahedral geometry (Fig. 1 and Table 1) with the two additional neighbours O1 and O3 with Zn-ligad distances of 2.711 (2) and 2.717 (2) Å, respectively. Each 5-aminoisophthalate anion acts as a µ_3_-bridge. So in the structure of title complex, every 5-aminoisophthalate anion links three zinc ions and every zinc ion bridges three 5-aminoisophthalate anions. This kind of connection proceeds infinitely to form a layer (Fig. 2). Whithin the crystal structure, there are N—H···O hydrogen bonds (Table 2).

### Experimental

Reaction mixture of zinc nitrate hexahydrate (29.7 mg, 0.1 mmol), 5-aminoisophthalic acid (18.1 mg, 0.1 mmol), imidazole (6.81 mg, 0.1 mmol), and potassium hydroxide (11.2 mg, 0.2 mmol) in 8 mL H_2_O was sealed in a 16 mL Teflon-lined stainless steel container and heated to(1) 453 K for 3 days. After cooling to the room temperature, colourless block crystals of the title complex were obtained.

### Refinement

The hydrogen atoms in all C atoms were located in geometrically idealized positions and constrained to ride on their parent atoms, with C—H = 0.93 Å and *U*_iso_(H) = 1.2*U*_eq_(C). The hydrogen atoms in N or O atoms can be found at reasonable positions in the difference Fourier maps and located there [*U*_iso_(H) = 1.2*U*_eq_(N or O)].

### Crystal data

**Table:**

[Zn(C_8_H_5_NO_4_)(C_3_H_4_N_2_)]·0.25H_2_O	*F*(000) = 642
*M**_r_* = 317.09	*D*_x_ = 1.768 Mg m^−^^3^
Monoclinic, *P*2_1_/*c*	Mo *K*α radiation, λ = 0.71073 Å
Hall symbol: -P 2ybc	Cell parameters from 2609 reflections
*a* = 9.6239 (11) Å	θ = 2.6–28.0°
*b* = 10.1916 (11) Å	µ = 2.08 mm^−^^1^
*c* = 12.1927 (13) Å	*T* = 293 K
β = 95.146 (2)°	Block, colourless
*V* = 1191.1 (2) Å^3^	0.20 × 0.20 × 0.18 mm
*Z* = 4	

### Data collection

**Table:**

Bruker SMART APEXII CCD diffractometer	2340 independent reflections
Radiation source: sealed tube	1542 reflections with *I* > 2σ(*I*)
graphite	*R*_int_ = 0.038
phi and ω scans	θ_max_ = 26.0°, θ_min_ = 2.1°
Absorption correction: multi-scan (*SADABS*; Sheldrick, 1996)	*h* = −11→11
*T*_min_ = 0.681, *T*_max_ = 0.706	*k* = −12→12
6327 measured reflections	*l* = −14→13

### Refinement

**Table:**

Refinement on *F*^2^	Primary atom site location: structure-invariant direct methods
Least-squares matrix: full	Secondary atom site location: difference Fourier map
*R*[*F*^2^ > 2σ(*F*^2^)] = 0.048	Hydrogen site location: inferred from neighbouring sites
*wR*(*F*^2^) = 0.098	H-atom parameters constrained
*S* = 1.09	*w* = 1/[σ^2^(*F*_o_^2^) + (0.04*P*)^2^] where *P* = (*F*_o_^2^ + 2*F*_c_^2^)/3
2340 reflections	(Δ/σ)_max_ < 0.001
178 parameters	Δρ_max_ = 0.56 e Å^−^^3^
0 restraints	Δρ_min_ = −0.57 e Å^−^^3^

### Special details

**Table:**

Geometry. All e.s.d.'s (except the e.s.d. in the dihedral angle between two l.s. planes) are estimated using the full covariance matrix. The cell e.s.d.'s are taken into account individually in the estimation of e.s.d.'s in distances, angles and torsion angles; correlations between e.s.d.'s in cell parameters are only used when they are defined by crystal symmetry. An approximate (isotropic) treatment of cell e.s.d.'s is used for estimating e.s.d.'s involving l.s. planes.
Refinement. Refinement of *F*^2^ against ALL reflections. The weighted *R*-factor *wR* and goodness of fit *S* are based on *F*^2^, conventional *R*-factors *R* are based on *F*, with *F* set to zero for negative *F*^2^. The threshold expression of *F*^2^ > σ(*F*^2^) is used only for calculating *R*-factors(gt) *etc*. and is not relevant to the choice of reflections for refinement. *R*-factors based on *F*^2^ are statistically about twice as large as those based on *F*, and *R*- factors based on ALL data will be even larger.

### Fractional atomic coordinates and isotropic or equivalent isotropic displacement parameters (Å^2^)

**Table:**

	*x*	*y*	*z*	*U*_iso_*/*U*_eq_	Occ. (<1)
C1	0.6575 (4)	0.3910 (3)	0.9523 (3)	0.0326 (9)	
C2	0.6177 (4)	0.3912 (4)	0.8394 (3)	0.0351 (9)	
H2	0.6037	0.3123	0.8016	0.042*	
C3	0.5989 (4)	0.5098 (3)	0.7836 (3)	0.0320 (8)	
C4	0.6275 (4)	0.6274 (3)	0.8374 (3)	0.0346 (9)	
H4	0.6188	0.7060	0.7987	0.042*	
C5	0.6696 (4)	0.6277 (3)	0.9507 (3)	0.0330 (9)	
C6	0.6827 (4)	0.5094 (4)	1.0076 (3)	0.0361 (9)	
H6	0.7084	0.5093	1.0830	0.043*	
C7	0.6734 (4)	0.2659 (4)	1.0171 (3)	0.0359 (9)	
C8	0.6970 (4)	0.7532 (4)	1.0138 (3)	0.0350 (9)	
C9	0.9582 (5)	0.1183 (4)	1.2027 (4)	0.0439 (11)	
H9	0.9093	0.1936	1.2188	0.053*	
C10	1.0136 (5)	−0.0654 (4)	1.1370 (3)	0.0441 (10)	
H10	1.0093	−0.1433	1.0972	0.053*	
C11	1.1245 (5)	−0.0237 (4)	1.2012 (4)	0.0449 (10)	
H11	1.2095	−0.0667	1.2149	0.054*	
N1	0.5567 (3)	0.5092 (3)	0.6679 (2)	0.0355 (7)	
H1B	0.4983	0.4405	0.6509	0.043*	
H1A	0.5065	0.5794	0.6488	0.043*	
N2	0.9069 (3)	0.0238 (3)	1.1382 (3)	0.0353 (7)	
N3	1.0886 (4)	0.0935 (3)	1.2423 (3)	0.0438 (9)	
H3	1.1418	0.1496	1.2921	0.053*	
O1	0.6978 (3)	0.2682 (2)	1.1177 (2)	0.0401 (7)	
O2	0.6629 (3)	0.1581 (2)	0.9615 (2)	0.0339 (6)	
O3	0.7375 (3)	0.7512 (2)	1.1132 (2)	0.0438 (7)	
O4	0.6761 (3)	0.8610 (2)	0.9600 (2)	0.0384 (7)	
O1W	0.0000	0.0000	0.5000	0.047 (2)	0.50
H1X	0.0422	0.0359	0.4495	0.056*	0.25
H1Y	0.0453	0.0137	0.5620	0.056*	0.25
Zn1	0.71550 (5)	0.01074 (4)	1.06385 (4)	0.03453 (16)	

### Atomic displacement parameters (Å^2^)

**Table:**

	*U*^11^	*U*^22^	*U*^33^	*U*^12^	*U*^13^	*U*^23^
C1	0.034 (2)	0.0284 (18)	0.034 (2)	0.0003 (16)	−0.0028 (18)	−0.0004 (15)
C2	0.035 (2)	0.032 (2)	0.036 (2)	−0.0002 (16)	−0.0064 (17)	−0.0001 (16)
C3	0.0285 (19)	0.035 (2)	0.0310 (19)	0.0022 (16)	−0.0039 (15)	−0.0037 (16)
C4	0.040 (2)	0.029 (2)	0.034 (2)	−0.0010 (16)	−0.0003 (18)	0.0027 (15)
C5	0.034 (2)	0.032 (2)	0.031 (2)	0.0018 (15)	−0.0062 (17)	−0.0021 (15)
C6	0.038 (2)	0.036 (2)	0.032 (2)	0.0021 (16)	−0.0083 (16)	0.0001 (15)
C7	0.035 (2)	0.034 (2)	0.037 (2)	0.0038 (16)	−0.0038 (17)	−0.0024 (17)
C8	0.035 (2)	0.032 (2)	0.037 (2)	−0.0009 (15)	−0.0066 (18)	0.0025 (16)
C9	0.048 (3)	0.0297 (19)	0.051 (3)	0.0036 (18)	−0.014 (2)	−0.0107 (18)
C10	0.049 (3)	0.037 (2)	0.044 (3)	0.0067 (19)	−0.0099 (19)	−0.0063 (18)
C11	0.044 (2)	0.043 (2)	0.046 (3)	0.002 (2)	−0.0061 (19)	0.003 (2)
N1	0.0411 (18)	0.0330 (17)	0.0302 (17)	−0.0026 (14)	−0.0086 (13)	0.0019 (13)
N2	0.0377 (18)	0.0316 (16)	0.0345 (18)	0.0041 (14)	−0.0080 (14)	−0.0050 (14)
N3	0.041 (2)	0.0408 (19)	0.045 (2)	−0.0043 (16)	−0.0191 (17)	−0.0124 (15)
O1	0.0579 (19)	0.0300 (14)	0.0304 (16)	−0.0015 (12)	−0.0078 (13)	0.0032 (11)
O2	0.0494 (17)	0.0292 (13)	0.0213 (12)	0.0004 (12)	−0.0069 (12)	0.0014 (10)
O3	0.0579 (19)	0.0308 (14)	0.0385 (17)	0.0036 (13)	−0.0185 (14)	−0.0028 (12)
O4	0.0510 (18)	0.0314 (15)	0.0303 (14)	0.0001 (12)	−0.0095 (13)	−0.0026 (11)
O1W	0.050 (5)	0.056 (5)	0.034 (4)	0.001 (4)	−0.004 (4)	0.004 (4)
Zn1	0.0402 (3)	0.0300 (3)	0.0312 (3)	0.0001 (2)	−0.00893 (17)	0.0000 (2)

### Geometric parameters (Å, °)

**Table:**

C1—C6	1.394 (5)	C9—H9	0.9300
C1—C2	1.396 (5)	C10—C11	1.335 (6)
C1—C7	1.500 (5)	C10—N2	1.372 (5)
C2—C3	1.391 (5)	C10—H10	0.9300
C2—H2	0.9300	C11—N3	1.352 (5)
C3—C4	1.382 (5)	C11—H11	0.9300
C3—N1	1.432 (4)	N1—Zn1^i^	2.082 (3)
C4—C5	1.405 (5)	N1—H1B	0.9104
C4—H4	0.9300	N1—H1A	0.8832
C5—C6	1.391 (5)	N2—Zn1	1.983 (3)
C5—C8	1.503 (5)	N3—H3	0.9504
C6—H6	0.9300	O2—Zn1	1.989 (2)
C7—O1	1.229 (5)	O4—Zn1^ii^	1.998 (3)
C7—O2	1.290 (4)	O1W—H1X	0.8500
C8—O3	1.239 (5)	O1W—H1Y	0.8501
C8—O4	1.287 (4)	Zn1—O4^iii^	1.998 (3)
C9—N2	1.312 (5)	Zn1—N1^iv^	2.082 (3)
C9—N3	1.327 (5)		
			
C6—C1—C2	119.8 (3)	C11—C10—N2	110.1 (4)
C6—C1—C7	118.4 (3)	C11—C10—H10	125.0
C2—C1—C7	121.8 (3)	N2—C10—H10	125.0
C3—C2—C1	119.7 (3)	C10—C11—N3	106.3 (4)
C3—C2—H2	120.1	C10—C11—H11	126.9
C1—C2—H2	120.1	N3—C11—H11	126.9
C4—C3—C2	120.6 (3)	C3—N1—Zn1^i^	116.3 (2)
C4—C3—N1	119.9 (3)	C3—N1—H1B	110.0
C2—C3—N1	119.4 (3)	Zn1^i^—N1—H1B	105.0
C3—C4—C5	119.8 (3)	C3—N1—H1A	110.9
C3—C4—H4	120.1	Zn1^i^—N1—H1A	109.4
C5—C4—H4	120.1	H1B—N1—H1A	104.5
C6—C5—C4	119.6 (3)	C9—N2—C10	104.5 (3)
C6—C5—C8	118.6 (3)	C9—N2—Zn1	127.4 (3)
C4—C5—C8	121.8 (3)	C10—N2—Zn1	128.0 (3)
C5—C6—C1	120.3 (3)	C9—N3—C11	107.4 (3)
C5—C6—H6	119.8	C9—N3—H3	123.7
C1—C6—H6	119.8	C11—N3—H3	128.8
O1—C7—O2	122.7 (3)	C7—O2—Zn1	108.0 (2)
O1—C7—C1	120.7 (3)	C8—O4—Zn1^ii^	108.5 (2)
O2—C7—C1	116.6 (3)	H1X—O1W—H1Y	109.5
O3—C8—O4	122.3 (3)	N2—Zn1—O2	114.21 (12)
O3—C8—C5	120.8 (3)	N2—Zn1—O4^iii^	117.19 (12)
O4—C8—C5	117.0 (3)	O2—Zn1—O4^iii^	98.96 (10)
N2—C9—N3	111.7 (3)	N2—Zn1—N1^iv^	115.47 (13)
N2—C9—H9	124.1	O2—Zn1—N1^iv^	107.26 (12)
N3—C9—H9	124.1	O4^iii^—Zn1—N1^iv^	101.77 (12)
			
C6—C1—C2—C3	−2.2 (6)	C4—C3—N1—Zn1^i^	92.1 (4)
C7—C1—C2—C3	177.4 (4)	C2—C3—N1—Zn1^i^	−84.6 (4)
C1—C2—C3—C4	4.0 (6)	N3—C9—N2—C10	−0.6 (5)
C1—C2—C3—N1	−179.4 (4)	N3—C9—N2—Zn1	177.5 (3)
C2—C3—C4—C5	−3.0 (6)	C11—C10—N2—C9	0.9 (5)
N1—C3—C4—C5	−179.6 (4)	C11—C10—N2—Zn1	−177.2 (3)
C3—C4—C5—C6	0.3 (6)	N2—C9—N3—C11	0.1 (5)
C3—C4—C5—C8	−177.8 (4)	C10—C11—N3—C9	0.5 (5)
C4—C5—C6—C1	1.5 (6)	O1—C7—O2—Zn1	−6.9 (5)
C8—C5—C6—C1	179.6 (4)	C1—C7—O2—Zn1	171.8 (3)
C2—C1—C6—C5	−0.6 (6)	O3—C8—O4—Zn1^ii^	−1.3 (5)
C7—C1—C6—C5	179.8 (4)	C5—C8—O4—Zn1^ii^	178.6 (3)
C6—C1—C7—O1	5.4 (6)	C9—N2—Zn1—O2	59.6 (4)
C2—C1—C7—O1	−174.2 (4)	C10—N2—Zn1—O2	−122.8 (3)
C6—C1—C7—O2	−173.3 (3)	C9—N2—Zn1—O4^iii^	174.6 (3)
C2—C1—C7—O2	7.1 (6)	C10—N2—Zn1—O4^iii^	−7.7 (4)
C6—C5—C8—O3	3.8 (6)	C9—N2—Zn1—N1^iv^	−65.5 (4)
C4—C5—C8—O3	−178.1 (4)	C10—N2—Zn1—N1^iv^	112.2 (3)
C6—C5—C8—O4	−176.1 (4)	C7—O2—Zn1—N2	−58.7 (3)
C4—C5—C8—O4	2.0 (6)	C7—O2—Zn1—O4^iii^	176.0 (3)
N2—C10—C11—N3	−0.8 (5)	C7—O2—Zn1—N1^iv^	70.6 (3)

Symmetry codes: (i) *x*, −*y*+1/2, *z*−1/2; (ii) *x*, *y*+1, *z*; (iii) *x*, *y*−1, *z*; (iv) *x*, −*y*+1/2, *z*+1/2.

### Hydrogen-bond geometry (Å, °)

**Table:**

*D*—H···*A*	*D*—H	H···*A*	*D*···*A*	*D*—H···*A*
N1—H1B···O4^v^	0.91	2.21	3.018 (4)	147.
N1—H1A···O2^vi^	0.88	2.17	2.943 (4)	146.
N3—H3···O3^vii^	0.95	1.88	2.822 (4)	174.

Symmetry codes: (v) −*x*+1, *y*−1/2, −*z*+3/2; (vi) −*x*+1, *y*+1/2, −*z*+3/2; (vii) −*x*+2, *y*−1/2, −*z*+5/2.
